# Case Department

**Published:** 1889-07

**Authors:** 


### CASE DEPARTMENT.

Luxation of Perforatus Tendon from Os Calcis. Recovery.—Bay gelding 8 years old, spirited farm horse, with a habit of rolling after work when first put in stable. Last March while rolling, he struck his hock against the wall and produced capped hock. He was treated by a "Vet." for several months without causing reduction of the swelling, when in September the owner asked him to lance it, which he did, cutting the attachment of the perforatus on the inside, letting out the synovia and allowing the perforatus to slip off of the os calcis externally, the horse becoming apparently worthless as he could hardly walk. I first saw him December 5th, 1888. Condition: Temperature, respiration and pulse normal and general condition good. The incision had healed. I could replace tendon on point of calcis, but as soon as leg was lifted the tendon would slip off again. There was a large synovial tumor on inside of hock opposite and anterior to flexor tendon which would become more posterior where tendon slips off calcis.

Dec. 10. I evacuated the synovia by means of trocar, size of canula 1/8 inch.

Dec. 12. I again evacuated and injected tr. iodine ʒi; then I put on a truss made of spring steel 1/4x3/4 inch, two pieces flattened out, one opposite flexor on outside, and the other anterior to flexor on inside. I then rivetted plates on the flattened parts and over these harness leather laced both above and below hock, ends of truss being long enough to protrude back of hock, so that rubber bandages could be put on to get the required pressure.

Dec. 15. Temperature, respiration and pulse normal, eating all right; standing with leg lifted; no swelling. The flexor in place but slips off as soon as truss is removed. I replaced it again.

Dec. 19. Still no swelling but standing with leg lifted. I think this is due to the pressure of truss as there is no fever. Placed in box stall; no slings used.

Dec 22. Truss torn off previous night by being caught in side of stall. It is now much swollen. Bathed with hot water, then replaced truss, first putting on roller bandage as truss is making an ulcer. Does not bear any weight on foot.

Dec. 26. Hock swollen a great deal but stands on leg more, some pus is discharging from ulcer which is superficial. Cannot keep truss on as he knows how to rub it off.

Dec. 29. Discontinued use of truss. Ordered hock bathed with soap liniment and fusil oil equal parts. Stands on one leg which is much swollen and hard.

Jan. 1. Not so much swollen; walks with very little lameness. Owing to the swelling it is difficult to say whether tendon is in place. The horse does not have that peculiar twist of leg in walking as before; stands on leg now.

Jan. 11. Swelling reduced fully one-half; scarcely any lameness. A large superficial ulcer on outside where truss pressed. No discharge or supperation at point of injection of iodine. Has not been allowed to lie down until to-day. Ordered application of tr. belladonna and iodine.

Jan. 18. Walks all right; swelling going down; ulcers healing.

March 25. Horse has been driven and worked without showing lameness. Point of hock swollen about twice the natural size, hard and with no point of fluctuation, but composed of dense fibrous tissue. Tendon in normal place with no tendency to slip off calcis.

W. H. Ridge, V. M. D.,

*Trevor, Pa.*

---

I. Prolonged Gestation in a Mare.—I have a mare thirty years of age who has been a regular breeder for the past six years. The last colt dropped was carried fourteen months, as she was mounted but once on the ninth day after dropping a filly. The last colt was strong, vigrous and large, having been sired by a standard bred trotter.

II. Cystic Tumor Containing Teeth.—While at Brock, Nebraska, I was called to remove a soft fluctuating tumor, about the size of a hen's egg, at the base and anterior aspect of a mare's ear. I made an incision and in opening pus and what proved to be a temporary tooth dropped out. The sac also contained a permanent tooth. I was compelled to use my large tooth forceps to extract it from the parietal bone, after which I removed the balance of the sac that contained the pus and other temporary teeth. The wound soon healed.

Another case similar to above was in a filly of mine. She was born with a small fluctuating tumor at base of ear, about the size of a pigeons egg. During the first summer it broke and continued to discharge yellow pus. When she was a yearling I opened the tumor and observed a tooth which I extracted. It healed, but has since broke and discharged pus several times. She is now nearly three years old and another tooth can now very plainly be felt at the same place, which I will probably extract this coming summer.

III. UTERO-INTESTINAL FISTULA.—Last November I was called to see a coming three year old heifer, and found her greatly emaciated and seemingly to be suffering from chronic diarrhœa. The history of the case is as follows: The June previous she was expected to have a calf. Labor pains came but she made no delivery. Her bag was full as usual in such cases. She made no presentation and was not assisted, but allowed to mope about, her bag dried up, an offensive discharge came from her, and after awhile her abdomen reduced in size and she became greatly reduced in flesh. On examination per vigina, I found the os very solid and rigid, and could introduce but two fingers. I was unable to dilate it in the least, but could feel the end of dry dead bones. To assist in dilating I injected some warm water into the uterus through a catheter, holding my fingers in the os to prevent its return; after injecting about a quart of water I could feel something give and the water all pass around my fingers, and on injecting more water it passed as I supposed through a break in the side of the uterus into the abdominal cavity, and would most likely cause fatal results if allowed to remain, so I concluded to open through the floor of the abdomen and relieve the uterus of its contents, at the same time allowing an escape of whatever had passed into the abdominal cavity. On opening into the uterus it seemed to be drawn in a very rigid firm condition over what seemed to be a dry lot of bones with no hair or flesh. I also discovered, grave plumb-pits, sand, and whole kernels of corn packed away among the bones. At this juncture the animal was destroyed, and on further examination I found that the small intestines had become united to the uterus and had formed an opening into the uterus, and five inches from the entrance was an exit into the intestine again, hence showing where the corn and gravel came from, as all the manure had to pass through the uterus.

JAMES VINCENT, D.V.M.

*Shenandoah, Iowa,*

